# Protective role of complement factor H against the development of preeclampsia

**DOI:** 10.3389/fimmu.2024.1351898

**Published:** 2024-02-23

**Authors:** Hadida Yasmin, Chiara Agostinis, Miriam Toffoli, Tamali Roy, Silvia Pegoraro, Andrea Balduit, Gabriella Zito, Nicoletta Di Simone, Giuseppe Ricci, Taruna Madan, Uday Kishore, Roberta Bulla

**Affiliations:** ^1^ Immunology and Cell Biology Laboratory, Department of Zoology, Cooch Behar Panchanan Barma University, Cooch Behar, West Bengal, India; ^2^ Institute for Maternal and Child Health, IRCCS Burlo Garofolo, Trieste, Italy; ^3^ Department of Medical, Surgical and Health Science, University of Trieste, Trieste, Italy; ^4^ Department of Biomedical Sciences, Humanitas University, Milan, Italy; ^5^ Istituto di Ricovero e Cura a Carattere Scientifico (IRCCS) Humanitas Research Hospital, Milan, Italy; ^6^ Department of Innate Immunity, ICMR-National Institute for Research in Reproductive and Child Health (NIRRCH), Mumbai, India; ^7^ Department of Veterinary Medicine, U.A.E. University, Al Ain, United Arab Emirates; ^8^ Zayed Centre for Health Sciences, United Arab Emirates University, Al Ain, United Arab Emirates; ^9^ Department of Life Sciences, University of Trieste, Trieste, Italy

**Keywords:** factor H, complement system, pregnancy, placenta, preeclampsia, microvesicles

## Abstract

Pregnancy is an immunologically regulated, complex process. A tightly controlled complement system plays a crucial role in the successful establishment of pregnancy and parturition. Complement inhibitors at the feto-maternal interface are likely to prevent inappropriate complement activation to protect the fetus. In the present study, we aimed to understand the role of Factor H (FH), a negative regulator of complement activation, in normal pregnancy and in a model of pathological pregnancy, *i.e.* preeclampsia (PE). The distribution and expression of FH was investigated in placental tissues, various placental cells, and in the sera of healthy (CTRL) or PE pregnant women *via* immunohistochemistry, RT-qPCR, ELISA, and Western blot. Our results showed a differential expression of FH among the placental cell types, decidual stromal cells (DSCs), decidual endothelial cells (DECs), and extravillous trophoblasts (EVTs). Interestingly, FH was found to be considerably less expressed in the placental tissues of PE patients compared to normal placental tissue both at mRNA and protein levels. Similar results were obtained by measuring circulating FH levels in the sera of third trimester CTRL and PE mothers. Syncytiotrophoblast microvesicles, isolated from the placental tissues of PE and CTRL women, downregulated FH expression by DECs. The present study appears to suggest that FH is ubiquitously present in the normal placenta and plays a homeostatic role during pregnancy.

## 1 Introduction

Pregnancy is a delicate biological process where the maternal immune system undergoes several immunomodulatory changes involving both innate and adaptive immunity to ensure tolerance at the feto-maternal interface. Complement is a crucial player in the tightly regulated immunological mechanisms for successful implantation, placental development, and parturition (1–3), displaying also non-canonical functions. Several years after its discovery, the contribution of the complement system to a successful pregnancy has increasingly been recognised (4).

Complement is a pivotal component of humoral innate immunity, which is made up of over 50 fluid-phase, membrane-bound and intracellular proteins that participate in three independent but collaborative activation pathways: classical, lectin and alternative. These three pathways converge on the activation of common component C3, and on the generation of anaphylatoxins such as C3a, C4a, C5a, and C5a-desArg, opsonins such as, C3b, iC3b, C3d, C4b, and cytolytic membrane attack complex (MAC) (5). Complement activation must be tightly regulated since complement dysregulation or over-activation can turn it from a homeostatic to a pathological effector driving several inflammatory conditions.

Several studies have demonstrated that dysregulation of complement activation predisposes to the development of pregnancy-related complications, such as pre-term birth, spontaneous abortion, recurrent miscarriage, and preeclampsia (PE) (6–9). PE is a common pregnancy disorder characterized by high blood pressure with proteinuria or end-organ injury. It is characterized by abnormal placentation, which may be related to defective decidualization, implantation, and angiogenesis (10–12). Besides shallow trophoblast invasion and inadequate remodeling of uterine arteries, dysregulation of immune and non-immune system is also a focal point in the development of PE. Excessive increase in the apoptosis of villous trophoblast can also cause over-activation of complement, enhancing pro-inflammatory response at the feto-maternal interface as observed in the case of PE (13). Moreover, increased levels of complement components and their activation-dependent cleaved products, including C3a, C5a, and C5b-9 complex, have been reported in the circulation of PE women (14), even though their exact contribution to PE pathophysiology is not fully understood.

Complement activation is tightly controlled through several complement regulatory proteins, Factor H (FH) being one of them in pregnancy. FH is 155 kDa soluble protein. Its gene is located on human chromosome 1 at the RAC (regulators of complement activation) gene cluster (15–17). Under physiological conditions, FH levels in the plasma range widely from 116 to 562 μg/ml, depending on genetic and environmental factors (18). FH is the main negative regulator of the complement alternative pathway in solution phase, but it is also involved in the inhibition of complement amplification on target cells and in the extracellular matrix of host tissues. It is a versatile innate immune molecule that protects host tissues by attenuating C3b attached to self surfaces and by restricting the formation of MAC. FH regulates enzymatic cleavage of C3 via its decay accelerating activity as well as cofactor activity for factor I–mediated C3b cleavage (19–22). Thus, by acting as a central negative regulator of the alternative pathway, FH limits the complement-mediated tissue damage leading to several diseases (23–25). FH can bind to molecules/ligands exposed on apoptotic and necrotic cells, including DNA, histones, and annexin II (and possibly the altered glycocalyx), in order to minimize the downstream pro-inflammatory effects of complement activation and the release of autoantigens that would potentially trigger autoimmunity (26–28).

Recent reports suggest that the FH level rises in maternal circulation during the first trimester of normal pregnancy (7), whereas it appears to decrease in severe PE (29). The presence of FH in placental tissue is largely attributed to the circulating blood in the placental vessels; whether it is synthesized locally by the major cell types constituting the placenta has not been investigated. The current study investigates the expression of FH in serum, placental tissues as well as various placental cells in normal pregnancy. FH levels were also measured in PE women to establish its contribution to the mechanisms underlying complement-dependent PE pathophysiology.

## 2 Materials and methods

### 2.1 Human subjects and samples

For this study, multiple cohorts of pregnant women at different stages of pregnancy were included (**Supplementary Figure 1**). For each cohort, PE was defined as the new onset of hypertension (systolic blood pressure ≥ 140 mmHg or diastolic ≥ 90 mmHg) on at least two occasions within 24 hours, along with the new onset of proteinuria (≥ 300 mg in a 24-hour urine collection, 50 mg/mmol protein/creatinine ratio, or at least 2+ on dipstick testing following two consecutive measurements).

A first cohort of patients was enrolled at the Institute for Maternal and Child Health, IRCCS “Burlo Garofolo” (Trieste, Italy) between October 2007 and April 2009, as described by Di Lorenzo and colleagues (30). From this serum bank, we selected first trimester sera of women who subsequently developed PE (*n* = 20) and sera of healthy pregnant women (*n* = 20) matched for maternal age (32 ± 4).

The third-trimester sera cohort was collected at the IRCCS “Policlinico Agostino Gemelli” (Rome, Italy). In particular, PE patients (*n* = 31) and CTRL (*n* = 26) had singleton pregnancies with undetected fetal abnormality and were matched for gestation week (32.7 ± 3.7 vs. 32.9 ± 4.5) (**Table 1**). A random selection from this cohort of samples was made to obtain pooled sera for cell stimulation. Blood samples were centrifuged at 500 *xg* for 7 min and the serum was immediately stored at -80°C.

**Table 1:** Characteristics of the PE patients’ cohort enrolled at Institute “Policlinico Agostino Gemelli” (Rome, Italy).

	PE n=31	CTRL n=26
Gestation weeks	32.7 ( ± 3.7)	32.9 ( ± 4.5)
Newborn weight (g)	1732 ( ± 860)	n.d.
Hypertension	75%	0%
Proteinuria (g/L)	2.7 (± 4)	0%
IUGR	35%	0%
Delivery		
Cesarean	80%	n.d.
Vaginal	20%	n.d.
Pre-existing diseases		
Hypertension	10%	0%
APS	15%	0%
Others	10%	0%

Data are expressed as mean ± standard deviation and as a percentage. n.d., not defined, IUGR, intra uterine growth restriction; APS, antiphospholipid syndrome.

Tissue samples of first-trimester placenta and decidua were obtained from healthy women undergoing elective termination of pregnancy at 8-12 weeks of gestation at the Institute for Maternal and Child Health, IRCCS “Burlo Garofolo” (Trieste, Italy). Within the same institute, term placenta samples were collected from both PE patients (*n* = 7) and healthy women (*n* = 8). A portion of these samples was fixed in 10%v/v buffered formalin for histological investigations, while another portion was shredded, added to 5 mL TRIzol (ThermoFisher), and stored at -80°C for total mRNA isolation.

The study was reviewed and approved by the Regional Ethical Committee of FVG (CEUR-2020-Os-156; Prot. 0022668/P/GEN/ARCS and CEUR-2019-Sper-17) and adhered to the principles of the Declaration of Helsinki. The written consent was obtained from all the participants.

Syncytiotrophoblast derived microvesicles (STBMs), prepared via dual-placental perfusion of PE and healthy placentae, were obtained from the Nuffield Department of Obstetrics and Gynaecology, John Radcliffe Hospital University of Oxford, United Kingdom, as previously described (31).

### 2.2 Cell isolation and culture

Endothelial and stromal cells were isolated from first trimester decidual biopsy specimens, as previously described, with minor modifications (32, 33). Briefly, decidual tissues were digested with 0.25% trypsin (Sigma-Aldrich) and 50 μg/mL DNase I (Roche, Milan, Italy) in PBS, overnight at 4°C, and then treated with 3 mg/mL collagenase type I (Worthington Biochemical, DBA, Milano, Italy), for 30 min at 37°C. Following Ficoll-Paque Plus density gradient (GE Healthcare, Euroclone, Milan, Italy) centrifugation, decidual human endothelial cells (DECs) were isolated by positive selection using Dynabeads M-450 (Dynal, Oslo, Norway) coated with lectin Ulex europaeus 1 (Sigma-Aldrich). DECs were cultured in a flask pre-coated with 5 μg/cm^2^ fibronectin (Roche) using Human Endothelial Serum Free Medium (HESFM; Gibco, Life Technologies, Milan, Italy), supplemented with 20 ng/mL basic Fibroblast Growth Factor (bFGF; Immunological Sciences), 10 ng/mL Epidermal Growth Factor (EGF; Immunological Sciences), and penicillin (50 U/mL)/streptomycin (50 μg/mL) (PS; Sigma-Aldrich).

Decidual stromal cells (DSCs) were obtained by culturing the endothelial-negative cell fraction in RPMI plus 10% fetal bovine serum (FBS), without growth factors. Non-adherent cells were removed *via* extensive washing; adherent cells were used only when the resulting cell population was negative for CD14, CD45, Cytokeratin 8/18, von Willebrand Factor (vWF), or CD31. The purity of DECs and DSCs isolated from first trimester decidua was routinely assessed by cytofluorimetric and qPCR analysis, as previously reported (34).

Extravillous trophoblasts (EVTs) were purified from placental tissues incubated with Hanks’ Balanced Salt Solution (HBSS) containing 0.25% trypsin and 0.2 mg/mL DNase I (Roche) for 20 min at 37°C, following the published procedure (35). Cells were seeded in 25 cm^2^-flask pre-coated with 5 μg/cm^2^ fibronectin (Roche) in RPMI (Gibco, Life Technologies) supplemented with 10% FBS to remove non-adherent leucocytes and syncytiotrophoblasts, and used within 12 h. The purity of EVTs isolated from first trimester placenta was routinely assessed by immunofluorescence and qPCR analysis, as previously reported (36).

Human umbilical vein endothelial cells (HUVECs) were isolated from umbilical cords of healthy placentae, following a published protocol (37), and cultured in HESFM supplemented with 20 ng/mL EGF, 10 ng/mL bFGF, 1% PS, and 10% FBS. Immortalized human hepatocytes (IHH, kindly provided by Dr. Cristina Bellarosa, Italian Liver Foundation, Trieste, Italy) were cultured in Dulbecco’s modified Eagle medium (Gibco), supplemented with non-essential amino acids, 2mM L-glutamine (Gibco). 100 μg/mL of penicillin/streptomycin (Gibco), and 10% FBS at 37°C and 5% CO_2_.

### 2.3 Immunohistochemical analysis

Term placental tissues from PE and healthy women were fixed in 10% *v/v* buffered formalin, paraffin-embedded and stored at 4°C. Tissue sections of 3-4 µm were deparaffinized with xylene and rehydrated with decreasing concentrations of ethanol (100%, 95%, 70%) and dH_2_O. The antigen unmasking technique was performed using citrate buffer (pH 6), for 20 min at 98°C in thermostatic bath. After neutralization of the endogenous peroxidase activity with 3% *v/v* H_2_O_2_ for 5 min, sections were incubated with PBS + 2% bovine serum albumin (BSA) for 30 min to block non-specific binding. Next, rabbit anti-human FH polyclonal antibody (1:100, #PA5-83957, Invitrogen) was incubated at 4°C. Staining was revealed *via* anti-rabbit horseradish peroxidase (HRP)-conjugate (1:500, Sigma), incubated for 30 min at room temperature, and 3-amino-9-ethylcarbazole (AEC, Vector Laboratories). Sections were counterstained with Mayer Haematoxylin (DiaPath, Italy) and examined under a Leica DM 2000 optical microscope. Kidney was stained as positive control tissue. Images were collected using a Leica DFC 7000 T digital camera (Leica Microsystems, Wetzlar, Germany). To quantify the staining, we utilized an immunoreactive score (38), which is commonly used for immunohistochemical evaluation. For each slide, we blindly analyzed three different visual fields of the microscope, attributing the following scores: 0 = no color reaction, 1 = mild reaction, 2 = moderate reaction, 3 = intense reaction.

### 2.4 Real time quantitative PCR

Total RNA was extracted from placental tissues of PE and healthy (CTRL) women as well as from isolated placental cells. The isolation of RNA from tissue samples was carried out using Phenol-Chloroform extraction method. Placental cells (EVTs, DECs, and DSCs) and IHH were stimulated for 24 h with 10% sera of PE or CTRL women (5 pooled sera), 100 ng/mL TNF-α, 10 ng/mL IL-1β or 50 μg/mL STBMs derived from PE or CTRL women. After incubation, cells were lysed and the total RNA was isolated. For each placental cell type (EVTs, DECs or DSCs), the experiments were repeated using at least four different cell populations. Total RNA was isolated using an RNA purification kit (Norgen Biotek Company, Thorold, Canada). RNA quantification was performed *via* NanoDrop® spectrometer (NanoDrop). RNA was converted to cDNA using SensiFAST™ cDNA Synthesis Kit (Meridian Bioscience, Memphis, TN, USA).

For Real-Time quantitative PCR (RT-qPCR), SYBR Select Master Mix (Applied Biosystems, ThermoFisher) was used. The reaction was performed by the Rotor-Gene 6000 (Corbett, Explera), *via* 45 cycles of denaturation (10 sec at 95°C), and annealing (45 sec at 60°C, melting temperature of the primers). Following primers were used: *CFH* (forward 5’- ACC GTG AAT GTG ACA CAG ATG G-3’; reverse 5’- ATT CCC GAT CTG GTT CCA TTG C-3’), *GAPDH* (forward 5’-GAT CAT CAG CAA TGC CTC CT-3’; reverse 5’- GT GGT CAT GAG TCC TTC CA-3’). The mRNA expression level of FH was evaluated with a comparative quantification provided with Rotor Gene 1.7 Software (Corbett Research) and normalized with the expression of a housekeeping gene (*GAPDH*).

### 2.5 Western blot analysis

Placental STBMs, isolated from CTRL (*n* = 4) or PE patients (*n* = 4), were lysed. After boiling, 20 µg of STBMs, diluted in Laemmli buffer, were subjected to a 10% *v/v* SDS-PAGE under reducing conditions. Proteins were transferred to a nitrocellulose membrane using the wet system of Mini Blot Module (Thermo Fisher Scientific), at a constant voltage for 60 min. An hour of blocking step with 5% skimmed milk in Tris-Buffered Saline + Tween 20 (TBST; 10 mM Tris, pH 8.0, 150 mM NaCl, 0.5% Tween 20) was followed by an overnight incubation at 4°C with primary antibodies: anti- FH (1:1000; #PA5-83957, Invitrogen) and anti-actin (1:1000; sc-8432, Santa Cruz). The following day, the membrane was incubated with anti-rabbit LI-COR IRDye 800CW and anti-mouse LI-COR IRDye 680CW (1:10,000; LI-COR Biosciences, Lincoln, NE, USA), for 1 h at room temperature. The fluorescence intensity was acquired in the Odyssey® CLx near-infrared scanner (LI‐COR Biosciences, Lincoln, NE, USA) and normalized for anti-actin. Image acquisition, processing and data analysis were performed via Image Studio Ver 5.2 (LI-COR Biosciences).

### 2.6 Enzyme-linked immunosorbent assay

Human Complement FH DuoSet ELISA kit (#DY4779; R&D Systems, Inc., Minneapolis, Canada) was used to quantify FH in serum samples, following the manufacturer's instructions. The plate was read by the PowerWave X Microplate Reader (Bio-Tek Instruments) spectrophotometer at 450 nm.

### 2.7 Statistical analysis

Data obtained from *in vitro* cell stimulation assays were analyzed by unpaired non-parametric test (Wilcoxon) comparing resting and treated conditions, while data obtained from patient samples were analyzed by unpaired t-test comparing PE *vs* controls healthy. STBM analysis was statistically evaluated using Mann-Whitney test. Results were expressed as mean ± standard deviations. *p*-values (*p*) < 0.05 were considered statistically significant. All statistical analyses were performed using GraphPad Prism software 9.0 (GraphPad Software Inc., La Jolla, CA, USA).

## 3 Results

### 3.1 FH expression in placental tissues and cells in normal pregnancy

**Figure 1:**
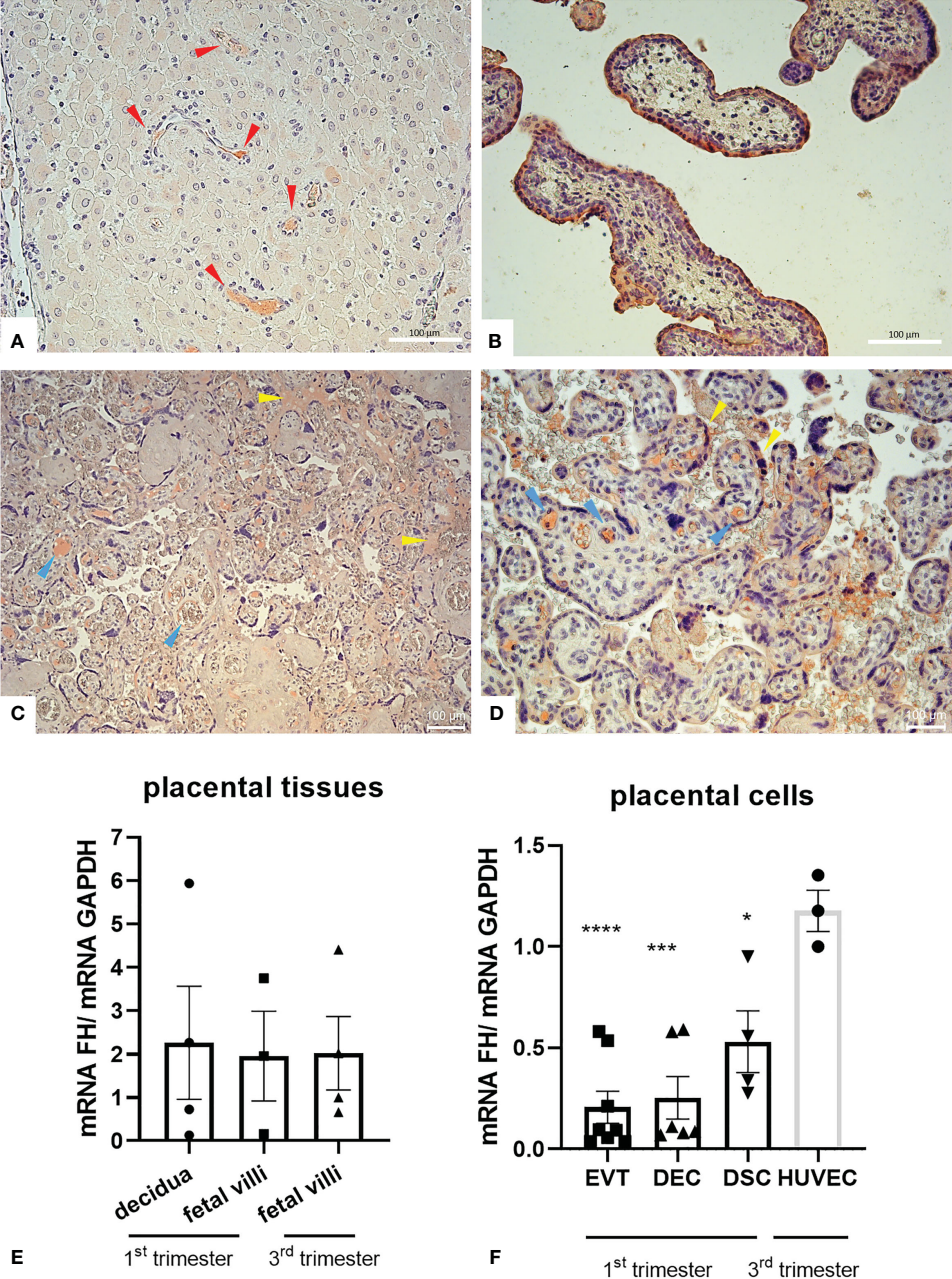
**(A–D)** Immunohistochemical analysis for the distribution of Factor H (FH) in placental tissues. First trimester [**(A, B)** *n* = 3] and term human placental [**(C, D)** *n* = 8] sections were stained with anti-FH polyclonal antibody. Staining was detected *via* 3-amino-9-ethylcarbazole (AEC) substrate chromogen. Nuclei were stained with Mayer’s Hematoxylin. The positivity for FH staining in first trimester placentae was particularly intense in decidual vessels (red arrows) and in the syncytiotrophoblast monolayer, whereas in villi vessels (yellow arrows) and in intervillous spaces and syncythium surface (blue arrows) for term placentae. Magnification, **(A, B, D)** 200x, **(C)** 200x. Scale bar, 100 µm. **(E, F)** mRNA expression levels of FH were measured by RT-qPCR in placental tissues **(E)** and placental cells **(F)** isolated from normal pregnancies. FH was expressed in all placental tissues (*n* = 4 first trimester decidua samples, *n* = 3 first trimester villous samples and *n* = 4 term villous samples) and placental cells examined, *i.e.*, extravillous trophoblast (EVT, *n* = 8), decidual endothelial cells (DECs, *n* = 6), and decidual stromal cells (DSCs, *n* = 4). FH gene expression was analyzed using HUVECs as a calibrator, since they showed the highest FH expression. *GAPDH* was used as a housekeeping gene to normalize gene expression results. Data are expressed as mean ± SD of two independent experiments performed in triplicate. *p < 0.05, ***p < 0.001, ****p < 0.0001, as compared to HUVECs (Mann-Whitney test).

To investigate the physiological role of FH in placenta, we first assessed the basal expression of FH in placental tissues and cells. We analyzed FH tissue distribution by immunohistochemistry (IHC) using first trimester and term placental sections with rabbit anti-human FH antibodies. As shown in **Figures 1A, B**, the positivity for FH staining in first trimester placentae was particularly intense in decidual spiral arteries and in the syncytiotrophoblast monolayer, whereas in third trimester placenta, FH was present in the inner of villi vessels and at the level of intervillous spaces (**Figures 1C, D**). In order to confirm the local synthesis of FH, we performed RT-qPCR using placental tissue mRNA isolated from first and third trimester normal placenta. In placental tissues, FH expression was observed both in the first (*n* = 4) as well as third (*n* = 4) trimester samples derived from healthy pregnancies, where both the decidua and the fetal villi expressed FH (**Figure 1E**). The expression levels were widely variable among samples. No significant difference could be observed between first and third trimester.

To identify cells that were responsible for the local production of FH, we performed RT-qPCR on isolated first trimester placental cells (EVTs, DECs, and DSCs). FH gene expression was analyzed using HUVECs as a calibrator, since they showed the highest FH mRNA expression. FH was expressed at similar levels by all the three placental cell investigated (DSCs, DECs, and EVTs) (**Figure 1F**).

### 3.2 Higher levels of FH detected in normal placentae compared to PE placentae

**Figure 2:**
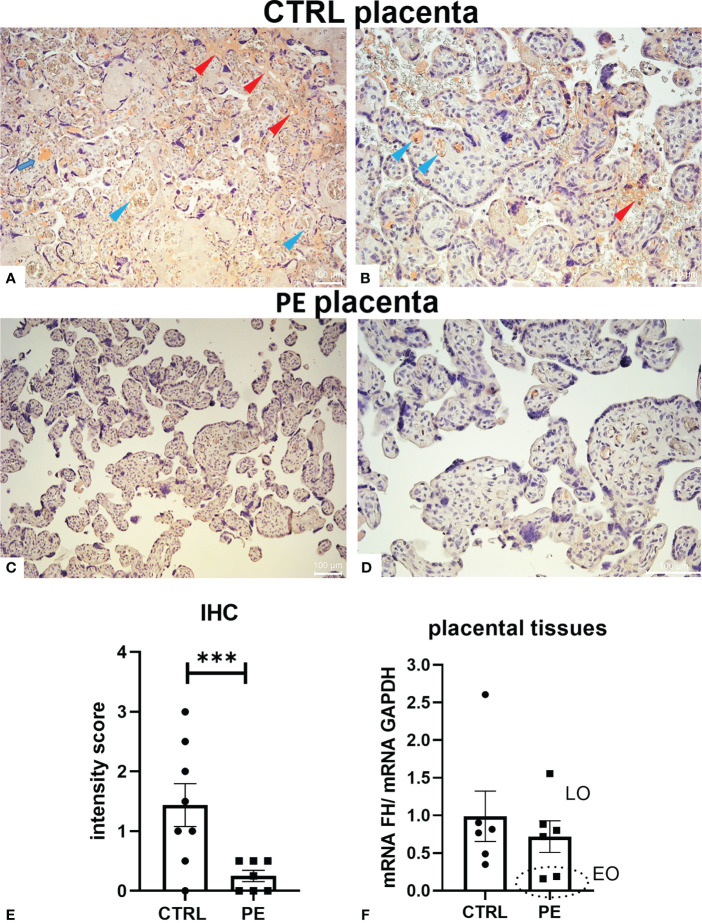
Localization of Factor H (FH) in placental tissues from normal (CTRL) and preeclamptic (PE) tissue. **(A–D)** The samples representative of CTRL [**(A)** original magnification 10 X; **(B)** original magnification 20X] or PE [**(C)** original magnification 10X; **(D)** original magnification 20 X] pregnancies were stained with anti-FH antibody. FH staining was detected in all the normal placental tissues, being mainly localized in fetal villi endothelium (blue arrows) and intervillous spaces (red arrows), whereas it was almost undetectable in PE placentae **(C, D)**. Staining was detected *via* 3-amino-9-ethylcarbazole (AEC) substrate chromogen. Nuclei were stained with Mayer’s Hematoxylin. Scale bars, 100 µm. **(E)** Quantitation of IHC staining using an immunoreactive score (PE *n* = 7, CTRL *n* = 8). For each slide, three different visual fields of the microscope, were analyzed giving a score as described in Materials and Methods section. ***p < 0.001 (t-test). **(F)** FH mRNA expression levels in term placental tissues of CTRL (*n* = 6) and PE [further divided into early onset, (EO, *n* = 2), and late onset, (LO, *n* = 4)] pregnancies were assessed by RT-qPCR. FH expression was lower in PE placentae compared to CTRL placentae; interestingly, EOPE samples exhibited minimal expression (marked with a circle). *GAPDH* was used as a housekeeping gene to normalize gene expression levels.

To better elucidate the role of FH in pregnancy, we first investigated its expression levels in a pathological condition that affects placental development, such as PE. Thus, we compared the local distribution of FH in CTRL and PE placental tissues by IHC. Placentae from CTRL women (*n* = 8) had higher FH expression than PE pregnancies (*n* = 7) (**Figure 2**, **Supplementary Figure 2**). FH was almost undetectable in PE placentae (**Figures 2C, D**), making it difficult to define its possible localization, even though villi vessels were slightly positive; FH in CTRL placentae was mainly localized in the fetal villous endothelium (blue arrows) and intervillous spaces (red arrows) (**Figures 2A, B**). The IHC quantitation demonstrated a significantly lower level of FH in PE placentae (**Figure 2E**). Our results were partially confirmed by RT-qPCR analysis of placental tissue. Although no statistical significance was reached, the results showed lower FH gene expression in PE placental tissue compared to CTRL placenta (**Figure 2F**). When dividing patients into late-onset (LO) and early-onset EOPE, we found that EOPE patients had the lowest FH levels (**Figure 2F**), suggesting the involvement of FH in PE-associated pathologic mechanisms.

### 3.3 Modulation of FH gene expression by sera of CTRL and PE women

**Figure 3:**
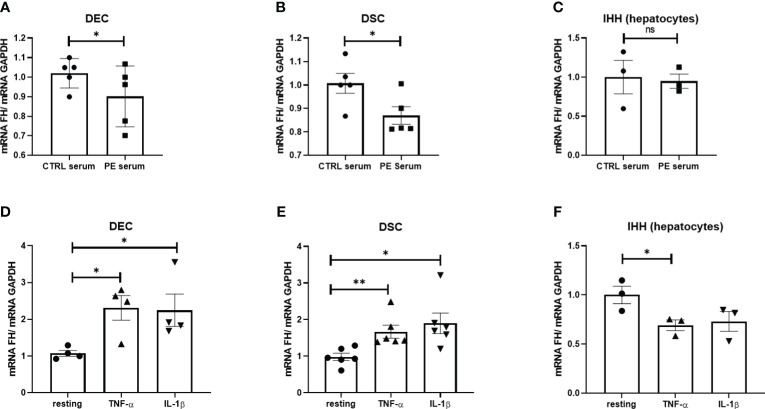
**(A–C)** Levels of Factor H (FH) mRNA expression in DECs **(A)**, DSCs **(B)** and IHH **(D)** following *in vitro* stimulation with serum from normal (CTRL) and PE women for 24 h. Pooled PE (*n* = 5) or CTRL (*n* = 5) serum samples were used. **(D–F)** Levels of FH mRNA expression in DECs **(D)**, DSCs **(E)** or IHH **(F)** stimulated with TNF-α or IL-1β for 24h. FH mRNA levels were significantly increased in DECs and DSCs after stimulation with TNF-α and IL-1β, whereas in IHH, FH transcripts were down-regulated with only TNF-α. Data are expressed as mean ± SD of two independent experiments performed in triplicate. *p < 0.05, **p < 0.01, as compared to resting (Mann-Whitney test). ns, non significant.

Based on previous observations, we hypothesized that microenvironmental factors, including pro-inflammatory cytokines present in the PE placentae, could be responsible for the decreased FH expression by placental cells. Thus, DECs and DSCs isolated from first trimester placentae were stimulated with a pool of 5 different CTRL or PE sera, and analyzed for FH mRNA expression levels. As shown in **Figures 3A, B**, FH expression was significantly reduced in DECs and DSCs following incubation with PE sera compared to cells incubated with matched CTRL sera. We also investigated the ability of PE sera to modulate FH expression using an immortalized hepatocyte cell line (IHH), but we did not observe any significant modulation of FH transcript levels (**Figure 3C**).

To determine which humoral factors in PE sera might be responsible for modulating FH expression, placental cells and IHH were stimulated with key pro-inflammatory cytokines known to be elevated in PE sera, such as TNF-α (39) and IL-1β (40). Surprisingly, none of these cytokines were able to induce a decrease in FH expression by the placental cells, but rather we observed their upregulation (**Figures 3D, E**). In contrast, IHH showed a significant downregulation of FH expression, especially after stimulation with TNF-α (**Figure 3F**).

Out of a range of immunomodulatory properties exerted by the placenta, the shedding of STBMs may contribute significantly to pregnancy success (41, 42). Interestingly, STBM levels are elevated in women with PE, possibly contributing to vascular endothelial dysfunction (43). Therefore, DECs were incubated for 24 h with STBMs isolated from the placenta of PE and CTRL women. Consistent with the earlier study, STBMs isolated from PE placentae were able to downregulate FH expression (**Supplementary Figure 3**); however, we were unable to obtain a statistically significant result.

### 3.4 Circulating levels of FH in healthy pregnant women are higher than in PE patients

To examine whether the observations gathered at the placental level might also reflect at the systemic level, circulating FH levels were measured by ELISA in the sera of PE women collected at the time of diagnosis and in the sera of matched CTRL women.

**Figure 4:**
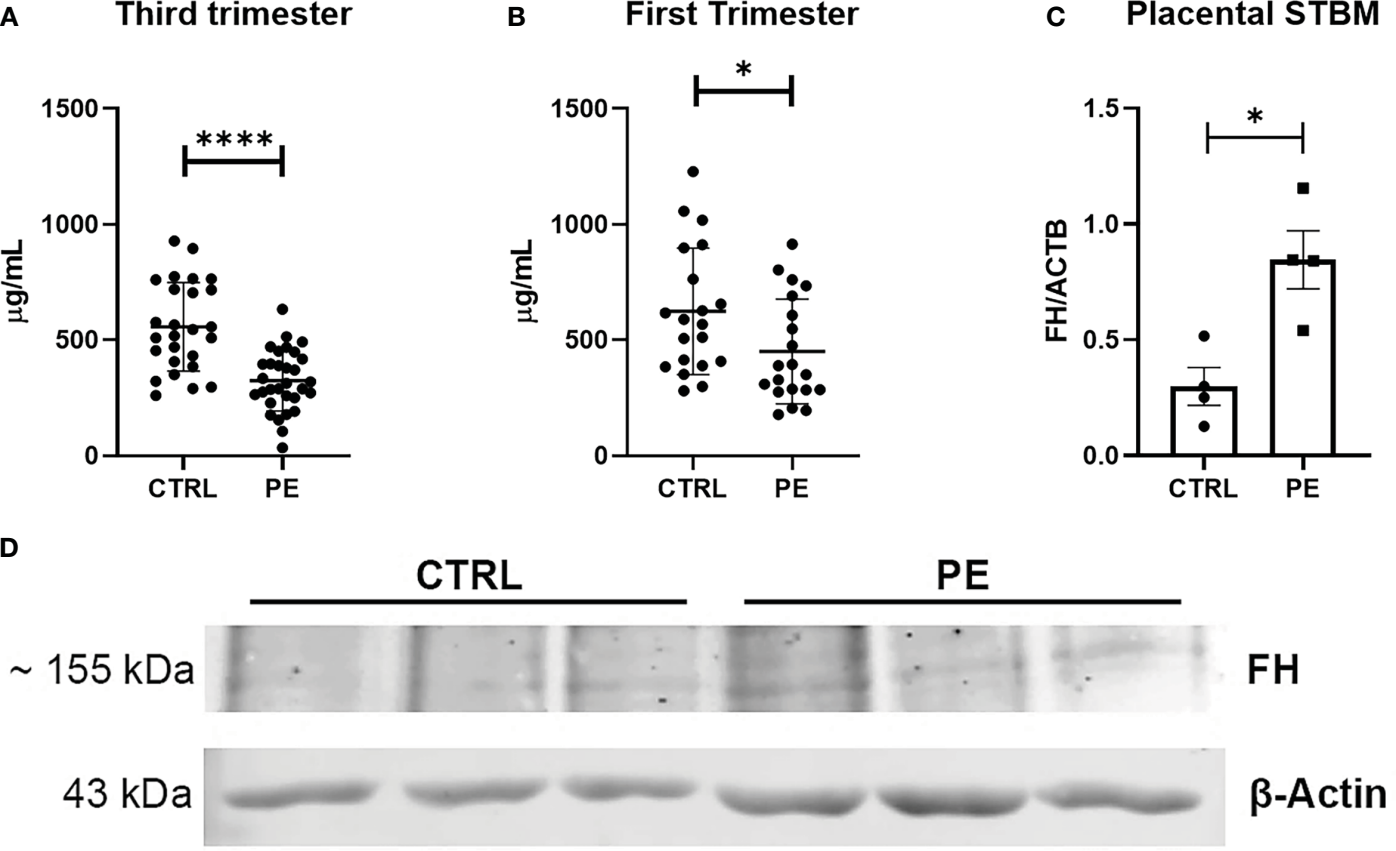
**(A, B)** Measurement of circulating Factor H (FH) levels in sera of normal (CTRL) and preeclamptic (PE) women, collected during third **(A)** or first **(B)** trimester, by ELISA. During both the first trimester and the third trimester lower levels of FH were observed in PE pregnancies compared to CTRL, showing a highly significant difference (*n* = 26 PE vs *n* =31 CTRL sera were compared for third trimester of pregnancy; *n* = 20 PE vs *n* = 20 CTRL for first trimester of pregnancy). *p < 0.05, ****p < 0.0001 (t-test). **(C, D)** Evaluation of FH deposition on placental STBMs via Western blot. STBMs derived from PE (*n* = 4) and CTRL (*n* = 4) were solubilized and separated by SDS-PAGE under reducing conditions. After transfer, the membrane was probed with anti-FH antibody and IRDye 800CW secondary antibody. Signal intensity was detected using an Odyssey CLx near-infrared scanner (LI-COR Biosciences, Lincoln, NE, USA). Image acquisition, processing and data analysis were performed with Image Studio 5.2 (LI-COR Biosciences). Histograms represent the mean ± SD of two independent experiments performed in duplicate. Beta-actin (ACTB) was used to normalize the results. *p < 0.05 (Mann-Whitney test).

In agreement with the results we obtained using placenta, we found lower circulating FH levels in the sera of PE women compared to CTRL (healthy) women (**Figure 4**). Therefore, we investigated whether the differences in FH levels between PE patients and CTRL women could be used to define FH as an early marker for PE. Thus, we quantified FH in serum samples collected between 11 and 13 weeks of gestation from pregnant women who later developed PE and in sera of matched CTRL women. Compared to PE, FH concentrations were significantly higher in the sera of CTRL women during the third trimester of pregnancy (**Figure 4A**, CTRL: 556.95 µg/mL ± SD 191.52 *vs* PE: 324.21 µg/mL ± SD 131.04), and, to a lesser extent, in the first trimester (**Figure 4B** CTRL: 624.06 µg/mL ± SD 273.05 *vs* PE: 450.97 µg/mL ± SD 226.16). Since a significant difference in FH levels was observed between PE and CTRL sera, we hypothesized that FH may bind to apoptotic bodies of the placenta. Therefore, we investigated the presence of FH deposition on STBMs, a method previously described by Tannetta et al. (44). We performed Western blot analysis using solubilized STBMs from PE (*n* = 4) and CTRL placentas (*n* = 4), and measured FH protein expression levels. As shown in **Figures 4C, D**, FH was present in STBMs from both PE placenta and CTRL, with significantly higher levels in PE.

## 4 Discussion

Pregnancy is a complex biological process in which the maternal immune system undergoes several changes that are necessary for the protection of the mother and the fetus from pathogenic challenges and for a successful delivery of the fetus (4). The complement system is critical for a successful pregnancy (1, 2), thus dysregulation along the complement cascade may play a major role in pregnancy-associated complications, including PE. In particular, complement regulators impose strict control on complement activation. FH, a negative regulator of complement alternative pathway, is a very multifaceted molecule which is able to bind a wide range of target ligands and fulfil different functions.

Aiming to investigate the role of FH in pregnancy, we first explored its expression pattern during different trimesters of the pregnancy. Our results confirmed a local production of FH at the feto-maternal interface, both in the decidua and in the villi, but showed no differences in FH transcript levels between the first and third trimester of pregnancy. IHC staining pattern was very different between the first trimester and term placenta: in first-trimester double-layered syncytium, FH staining was widespread; conversely, in term placenta monolayered villi, FH was present in maternal intervillous space and into the fetal villous vessels. Placental tissue may present intravillous fibrin deposits, which are expected to stain for FH (45). This is likely to represent the maternal FH, while the expected endothelial staining of villous vessels is the fetal FH; thus, the two display the expression of FH molecules of different origins.

To determine which placental cells were responsible for the local production of FH, we analyzed the cells isolated from first trimester placental tissue by RT-qPCR. All the placental cell types (DSCs, DECs, and EVTs) expressed FH to a similar degree, although the highest FH expression was observed in HUVECs, which we used as a positive control based on an earlier study by Rayes et al. (46).

Compared to PE, placentae from CTRL women had higher FH staining, which allowed us to conclude that FH was mainly localized in the fetal villous endothelium and intervillous spaces. Conversely, FH in PE placenta was almost undetectable. The presence of FH in healthy placenta has been previously reported with a significantly variable staining pattern, being uniformly localized in the syncytiotrophoblast layer in 70% of the samples, but not in the villous vessels (45). However, in agreement with our results, most of PE placentae remained negative for FH staining (45). Lokki and Heikkinen-Eloranta demonstrated intracellular localization of FH in the placental syncytiotrophoblast of PE placenta, and suggested that FH may exacerbate thrombotic microangiopathy in PE independent of complement activation (47). However, the existence of an association between FH and PE should be further confirmed by analyzing FH localization and synthesis *via* IHC and RT-qPCR using a greater number of cases, via an accurate clustering of the patients based on severity and pathogenesis of the disease. The results of Belmonte et al. demonstrated the involvement of the lectin pathway in complement deposition in PE placenta (48). This evidence is in accordance with our results that failed to detect FH deposition in PE placentae. However, we cannot exclude that FH staining was undetectable due to the damage and the dysmorphia typical of the PE villi.

Under physiological conditions, plasma FH levels can vary between 116 and 562 μg/mL depending on genetic and environmental factors, but they may increase in pregnant women (18). There are several reports about an increase in the circulating FH levels during pregnancy (7, 14, 49). Our study also suggests that soluble FH levels remain high during pregnancy both in the first and the third trimester. Interestingly, we observed a decrease in circulating FH levels when comparing PE with healthy pregnancy, particularly in the third trimester. Our results also highlighted a statistically significant difference between PE and healthy controls from the prospective study cohort. In the first trimester of pregnancy, patients who subsequently (after the 20^th^ week of gestation) developed PE had lower FH levels, indicating FH as a potential predictive marker of PE, although further studies are needed. Previous studies have already reported lower circulating levels of FH in PE patients (29, 50), in manner independent of the levels of anti-FH autoantibodies (50). In a prospective study, serum levels of FH were shown to increase in the middle and late stages of pregnancy, whereas FH was reduced in both EOPE and LOPE compared to control groups (29). However, these differences were not observed in the second and third trimesters of pregnancy. Another study pointed out that FH concentrations in the serum of PE patients were higher during the first trimester than in a normal pregnancy; subsequently, these values were similar to those in a normal pregnancy (7). Ari et al. found that serum C3 and FH levels in patients with HELLP (Hemolysis, Elevated Liver enzymes and Low Platelets) syndrome were not significantly different from those of PE patients and healthy pregnant women (51).

Insufficient levels of FH can be attributed to decreased FH expression due to mutations. Five rare variants (L3V, R127H, R166Q, C1077S, and N1176K) in the FH gene have been found to predispose to the occurrence of severe PE (52). Moreover, FH reduction can also be due to the presence of anti-FH autoantibodies, consumption of FH, or by the fact that FH is overwhelmed by a massive disease burden (53).

The observation of both local and systemic lower FH levels in PE led us to consider that microenvironmental factors may be responsible for reduced FH production by placental cells. After stimulation with pooled PE or control sera, we thus analyzed FH mRNA expression in DECs and DSCs. Our results showed a significantly reduced FH expression in cells stimulated with PE sera compared to control sera. The downregulation of FH gene may be explained by the particular conditions of PE, where the placenta is characterized by increased oxidative stress and complement activation. Thus, upregulation of the FH gene probably protects the cells by inhibiting complement activation under physiological conditions, while its downregulation can potentially lead to adverse pregnancy outcomes.

To identify the factors present in PE sera that might modulate FH expression, cells were incubated with pro-inflammatory cytokines known to be increased in PE women’s blood, such as TNF-α and IL-1β (39, 54–59). Previously, Daha’s group demonstrated that stimulation of HUVECs with IL-1 led to a decrease in FH expression (60). Unexpectedly, FH mRNA expression was increased in DECs and DSCs after stimulation with pro-inflammatory cytokines. Due to the multifactorial nature of PE (61), the *in vitro* stimulation with these two cytokines may likely be insufficient to mimic a PE-like scenario in placental cells. Moreover, the results obtained with IHH suggested that the selected amount of TNF-α (100 ng/mL) and IL-1β (10 ng/mL) was higher compared to their levels in PE sera. We can hypothesize differential levels of cytokine sensitivity between placental cells and hepatocytes. Since the potent pro-inflammatory properties of TNF-α and IL-1β can trigger excessive inflammation, and directly or indirectly, activate the complement system, placental cells upregulate FH expression possibly to control complement activation.

STBMs, released into the maternal circulation to ensure feto-maternal communication, are significantly increased in PE and can contribute to the local pro-inflammatory properties by carrying a variety of placental proteins into the maternal circulation (62). Tanetta et al. found 538 unique proteins in STBMs of PE, including several complement proteins and regulators (*e.g.*, C1q, C3, CD55, CD59, and vitronectin) (63). In another study, Baig et al. identified 25 differentially expressed proteins in PE, with CD59 being downregulated (64). Our results showed that STBMs, isolated from PE placentae, downregulated FH gene expression in DECs, suggesting that binding of STBMs to DECs may have an inhibitory effect on FH production. Surprisingly, we also found higher levels of FH in STBMs isolated from PE samples than in control samples. Despite this high proportion of FH in STBMs, complement activation is dysregulated/poorly controlled in PE. One possibility is that FH expressed by/on the placental cells is sequestered into STBMs, which may act as decoy substrates. A previous study in tumour models demonstrated that FH can successfully be carried by extracellular vesicles (65). Thus, FH may not be available to the cells for complement regulation, or alternatively, its ability to inhibit complement activation may be masked. In fact, it is widely recognized that FH must be in soluble form to be biologically effective, which is not the case in PE, consistent with the lower circulating FH levels observed in PE mothers when analyzing serum, tissues, and isolated cells. Therefore, it is important to investigate the immunomodulatory properties of FH-loaded STBMs in PE immunopathology.

## 5 Conclusions

Under physiological conditions, FH is locally expressed at the feto-maternal interface in all trimesters of pregnancy. However, it can be localized in syncytiotrophoblasts in the first trimester and in villous endothelium in the third trimester placenta. Decidual cells (DECs, EVTs, and DSCs) appear to synthesize FH. PE placentae are almost negative for FH staining. Circulating levels of FH are significantly lower in the PE sera compared to controls. The causes for this reduction could be multiple (*e.g.*, mutations, consumption, less synthesis). PE sera contain humoral factors that can cause downregulation of FH expression by placental cells, possibly due to the presence of STBMs highly loaded with FH in PE. Further studies are required to understand the mechanisms linking FH and PE onset.

## Data availability statement

The raw data supporting the conclusions of this article will be made available by the authors, without undue reservation.

## Ethics statement

The study was reviewed and approved by the Regional Ethical Committee of FVG (CEUR-2020-Os-156; Prot. 0022668/P/GEN/ARCS and CEUR-2019-Sper-17) and adhered to the principles of the Declaration of Helsinki. The studies were conducted in accordance with the local legislation and institutional requirements. The participants provided their written informed consent to participate in this study.

## Author contributions

HY: Data curation, Formal analysis, Investigation, Methodology, Project administration, Writing – original draft. CA: Formal analysis, Investigation, Methodology, Validation, Writing – original draft. MT: Investigation, Methodology, Software, Writing – review & editing. TR: Investigation, Methodology, Validation, Writing – review & editing. SP: Data curation, Investigation, Software, Writing – review & editing. AB: Data curation, Methodology, Software, Writing – review & editing. GZ: Formal analysis, Methodology, Software, Writing – review & editing. ND: Data curation, Investigation, Methodology, Writing – review & editing. GR: Formal analysis, Investigation, Methodology, Writing – review & editing. TM: Conceptualization, Investigation, Writing – review & editing. UK: Conceptualization, Formal analysis, Resources, Supervision, Writing – review & editing. RB: Conceptualization, Funding acquisition, Investigation, Methodology, Project administration, Resources, Writing – original draft.

## Supplementary material

Supplementary Figure 1 Graphical description of the multiple cohorts of pregnant women at different stages of pregnancy.

Supplementary Figure 2 IHC analysis of FH in placental tissues from three normal (CTRL) and three preeclamptic (PE) tissues. Original magnification 10X. FH staining was detected in all the normal placental tissues (left panels), whereas it was almost undetectable in PE placentae (right panels). Staining was detected *via* 3-amino-9-ethylcarbazole (AEC) substrate chromogen. Nuclei were stained with Mayer’s Hematoxylin. Scale bars, 100 µm.

Supplementary Figure 3 Levels of FH mRNA expression in DECs stimulated for 24h with 50 μg/mL STBMs of PE or CTRL placentae. Data are expressed as mean ± SD of two independent experiments performed in triplicate.
