# Supplementary material for: Protective role of complement factor H against the development of preeclampsia
Source: Front Immunol. 2024 Feb 23;15:1351898. doi: 10.3389/fimmu.2024.1351898 (PMC10920295; doi:10.3389/fimmu.2024.1351898)
Supplement: Supplementary Figure 1 — Graphical description of the multiple cohorts of pregnant women at different stages of pregnancy. [file DataSheet_1.docx]

Supplementary Material to

**Protective role of Complement Factor H against the development of Preeclampsia**

Hadida Yasmin^1†^, Chiara Agostinis^2,†^, Miriam Toffoli^3^, Tamali Roy^1^, Silvia Pegoraro^2^, Andrea Balduit^2^, Gabriella Zito^2^, Nicoletta Di Simone^4,5^, Giuseppe Ricci^2,3^, Taruna Madan^6^, Uday Kishore^7*^, Roberta Bulla^8*^

^1^ Immunology and Cell Biology Laboratory, Department of Zoology, Cooch Behar Panchanan Barma University, Cooch Behar 736101, West Bengal, India

^2^ Institute for Maternal and Child Health, IRCCS Burlo Garofolo, Trieste, Italy

^3^ Department of Medical, Surgical and Health Science, University of Trieste, Trieste, Italy

^4^ Department of Biomedical Sciences, Humanitas University, Milan, Italy

^5^ Istituto di Ricovero e Cura a Carattere Scientifico (IRCCS) Humanitas Research Hospital, Milan, Italy

^6^ ICMR-National Institute for Research in Reproductive and Child Health (NIRRCH), Mumbai, India

^7^ Department of Veterinary Medicine, U.A.E. University, Al Ain, United Arab Emirates

^8^ Department of Life Sciences, University of Trieste, Trieste, Italy

^†^ These authors have contributed equally to this work and share first authorship.

^*^ Corresponding authors: [ukishore@hotmail.com](mailto:ukishore@hotmail.com); [rbulla@units.it](mailto:rbulla@units.it)

**Running Title**: Factor H in preeclampsia

**Keywords**: Factor H, complement system, pregnancy, placenta, preeclampsia

**Supplementary Figure 1**


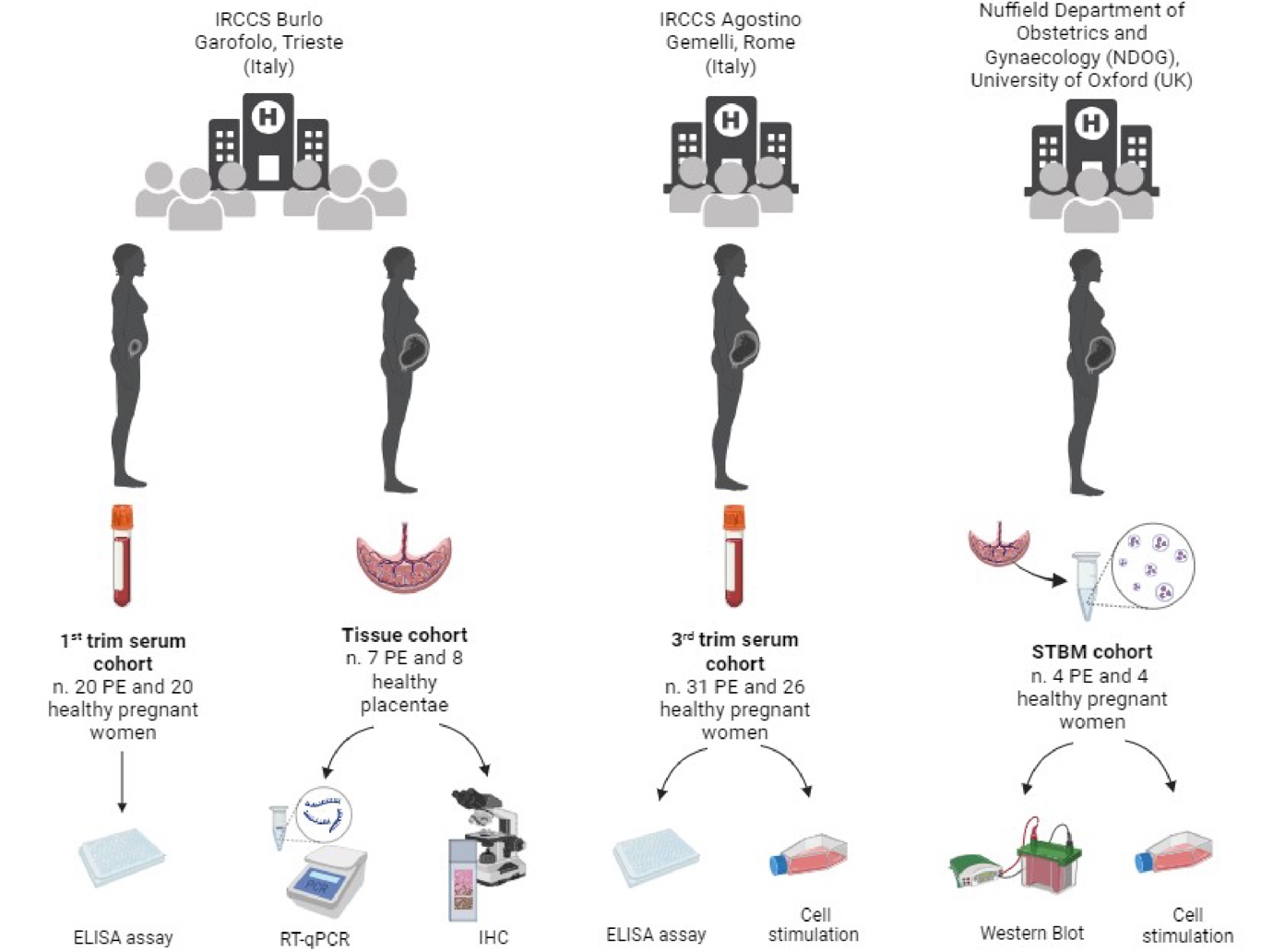


**Supplementary Figure 1**. Graphical description of the multiple cohorts of pregnant women at different stages of pregnancy.

**Supplementary Figure 2**


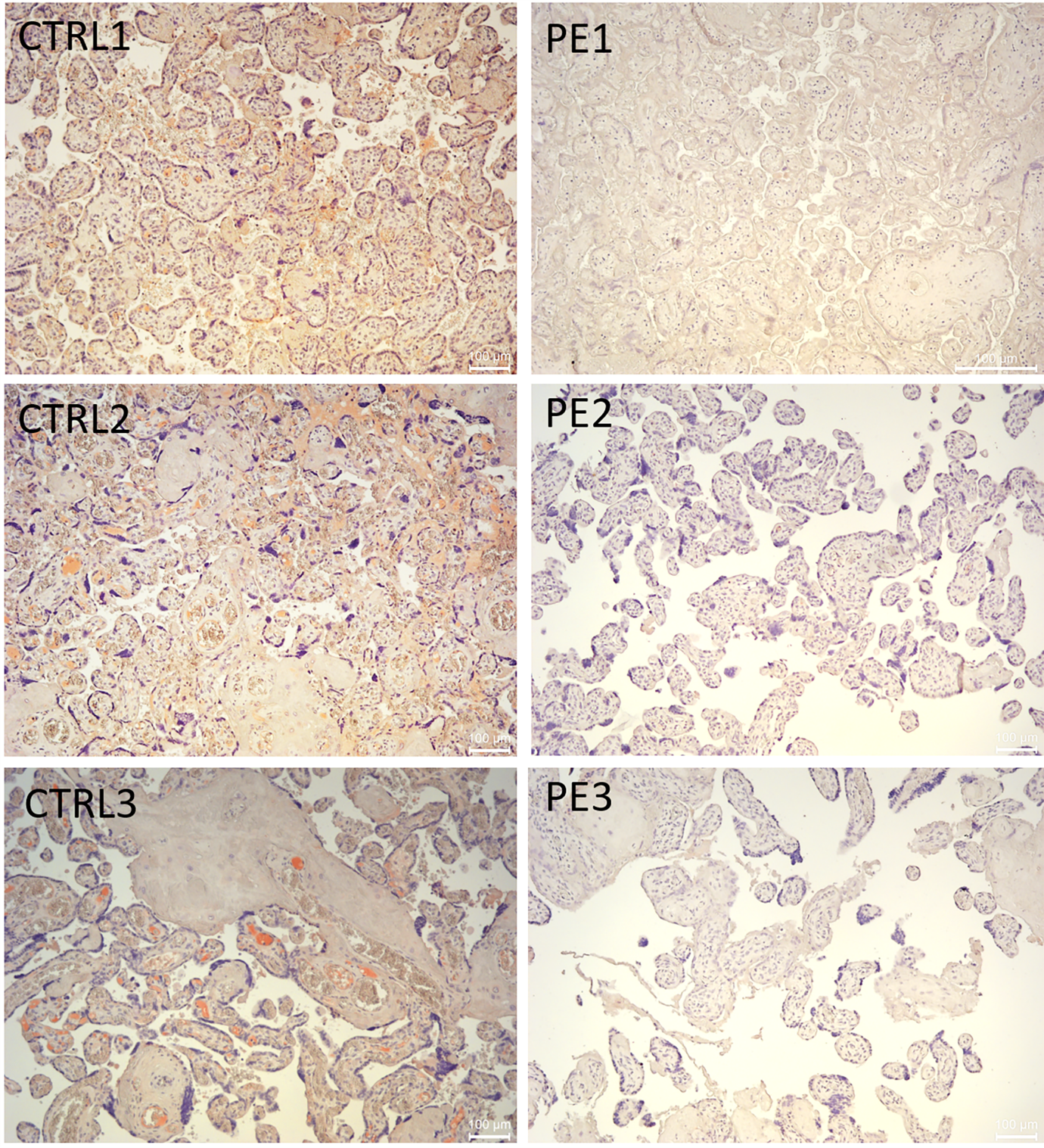


**Supplementary Figure 2.** IHC analysis of FH in placental tissues from three normal (CTRL) and three preeclamptic (PE) tissues. Original magnification 10X. FH staining was detected in all the normal placental tissues (**left panels**), whereas it was almost undetectable in PE placentae (**right panels**). Staining was detected *via* 3-amino-9-ethylcarbazole (AEC) substrate chromogen. Nuclei were stained with Mayer’s Hematoxylin. Scale bars, 100 µm.

**Supplementary Figure 3**


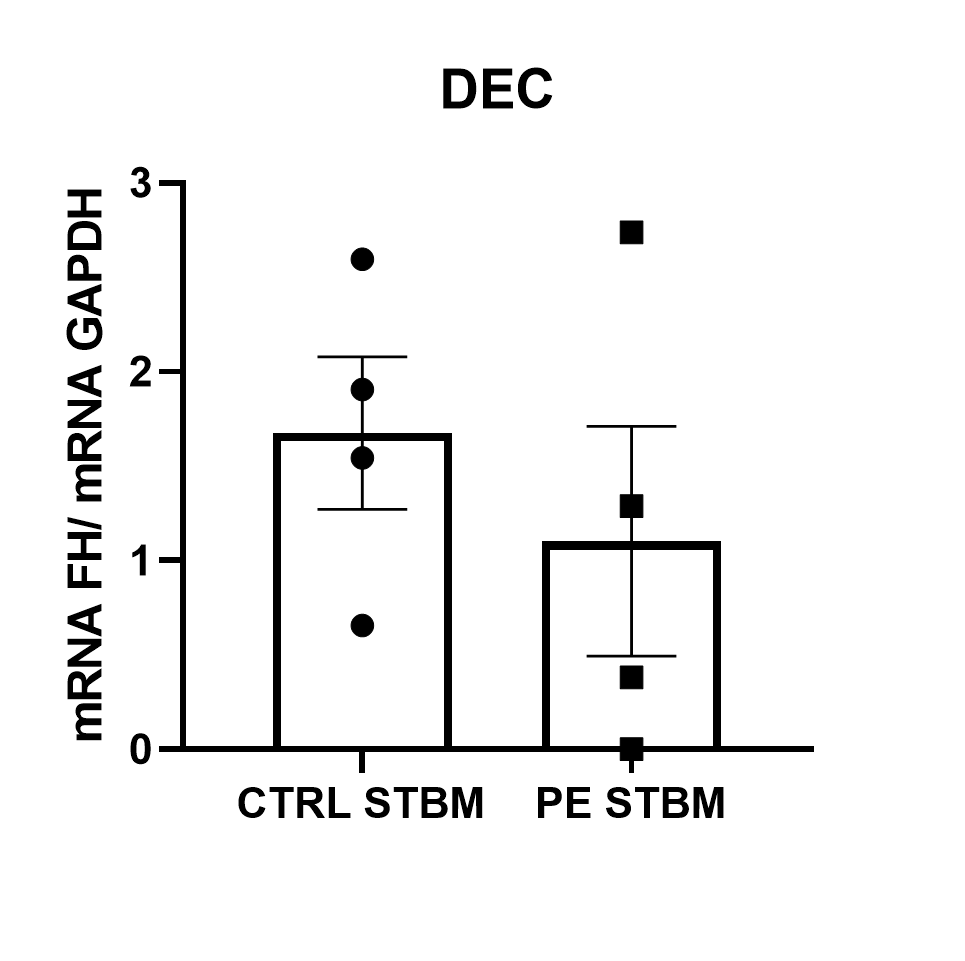


**Supplementary Figure 3.** Levels of FH mRNA expression in DECs stimulated for 24h with 50 μg/mL STBMs of PE or CTRL placentae. Data are expressed as mean ± SD of two independent experiments performed in triplicate.
